# Eriodictyol Protects Endothelial Cells against Oxidative Stress-Induced Cell Death through Modulating ERK/Nrf2/ARE-Dependent Heme Oxygenase-1 Expression

**DOI:** 10.3390/ijms160714526

**Published:** 2015-06-26

**Authors:** Seung Eun Lee, Hana Yang, Gun Woo Son, Hye Rim Park, Cheung-Seog Park, Young-Ho Jin, Yong Seek Park

**Affiliations:** 1 Department of Microbiology, School of Medicine, Kyung Hee University, Seoul 130-701, Korea; E-Mails: eunlee@khu.ac.kr (S.E.L.); yanghana@khu.ac.kr (H.Y.); warranty1010@naver.com (G.W.S.); hrsabina@naver.com (H.R.P.); pcs@khu.ac.kr (C.-S.P.); 2 Department of Physiology, School of Medicine, Kyung Hee University, Seoul 130-701, Korea; E-Mail: jinyh@khu.ac.kr

**Keywords:** flavonoid, eriodictyol, heme oxygenase-1, endothelial cells, oxidative stress, cell death

## Abstract

The pathophysiology of cardiovascular diseases is complex and may involve oxidative stress-related pathways. Eriodictyol is a flavonoid present in citrus fruits that demonstrates anti-inflammatory, anti-cancer, neurotrophic, and antioxidant effects in a range of pathophysiological conditions including vascular diseases. Because oxidative stress plays a key role in the pathogenesis of cardiovascular disease, the present study was designed to verify whether eriodictyol has therapeutic potential. Upregulation of heme oxygenase-1 (HO-1), a phase II detoxifying enzyme, in endothelial cells is considered to be helpful in cardiovascular disease. In this study, human umbilical vein endothelial cells (HUVECs) treated with eriodictyol showed the upregulation of HO-1 through extracellular-regulated kinase (ERK)/nuclear factor erythroid 2-related factor 2 (Nrf2)/antioxidant response element (ARE) signaling pathways. Further, eriodictyol treatment provided protection against hydrogen peroxide-provoked cell death. This protective effect was eliminated by treatment with a specific inhibitor of HO-1 and RNA interference-mediated knockdown of HO-1 expression. These data demonstrate that eriodictyol induces ERK/Nrf2/ARE-mediated HO-1 upregulation in human endothelial cells, which is directly associated with its vascular protection against oxidative stress-related endothelial injury, and propose that targeting the upregulation of HO-1 is a promising approach for therapeutic intervention in cardiovascular disease.

## 1. Introduction

Oxidative stress is known as a major contributor to endothelial dysfunction and one of the main causes of tissue damage in the endothelium [1]. Endothelial dysfunction is a key precursor to the development of vascular disease [2]. Oxidative stress induces expression of phase II-detoxifying and antioxidant enzymes [3], which are involved in the preservation of the cellular redox homeostasis and the elimination of toxic xenobiotics [4]. Heme oxygenase-1 (HO-1), a well-known phase II enzyme, is a cytoprotective, rate-limiting enzyme involved in heme degradation [5]. Upregulation of HO-1 expression plays an important role in the preservation of homeostasis in the face of oxidative damage, and it has been therapeutically implicated in various disorders [6].

Several studies have shown that the intake of polyphenols present in vegetables and fruits induces health benefits in humans. Flavonoids are polyphenolic compounds that are regularly consumed via the human diet; These compounds have known biological effects such as in the prevention of various disorders, including cardiovascular disease, due to their anti-inflammatory and antioxidant properties [7,8,9]. Furthermore, they can regulate several cell signaling pathways and stimulate the expression of phase II enzymes [10].

**Figure 1:**
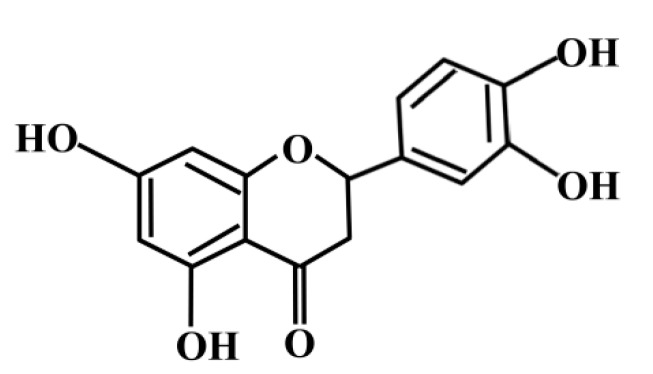
Chemical structures of the flavonoid eriodictyol (30,40,5,7-tetrahydroxyflavanone).

Eriodictyol is a flavonoid that is distributed in common fruits and vegetables, especially citrus fruits such as lemon [11,12]. It exhibits beneficial biological properties, including antioxidant and anti-inflammatory effects [13,14]. The structural differences of flavonoids such as the hydroxyl patterns of A- and B-rings and of the presence 2,3-unsaturationin conjugation with a 4-oxo group in the C-ring affect their beneficial biological properties [15]. The radical scavenging activities associated with the structure of eriodictyol protect against oxidative stress (Figure 1) [16]. Supplementation of the diet with lemon-derived flavonoids significantly suppresses oxidative stress in the liver, serum, and kidney of diabetic rats [17]. Recent studies have shown that eriodictyol prevents early retinal and plasma abnormalities and defends retinal cells against oxidative damage in diabetic rats [16,18,19]. Furthermore, several studies have indicated that eriodictyol has immunomodulatory effects, including inhibition of nitric oxide (NO) production by blockage of NF-κB activation as well as mitogen-activated protein kinase (MAPK) phosphorylation in macrophages and pro-inflammatory cytokine production through p38 MAPK, extracellular signal-regulated kinase (ERK)-, c-Jun N-terminal kinase (JNK)-, cyclooxygenase-2 (COX-2)-, and cluster of differentiation 14 (CD14)-dependent signaling pathways [20,21]. Moreover, eriodictyol has been shown to exhibit protective effects against oxidative toxicity in neuron-like cells [22]. However, the detailed molecular mechanism and effect of HO-1 upregulation in eriodictyol-stimulated endothelial cells have not been investigated.

In this study, we explored the possible cytoprotective effect of eriodictyol through ERK/Nrf2/ARE-mediated HO-1 upregulation in human endothelial cells.

## 2. Results

### 2.1. Effects of Induction of Heme Oxygenase-1 (HO-1) Expression by Eriodictyol in Human Umbilical Vein Endothelial Cells (HUVECs)

**Figure 2:**
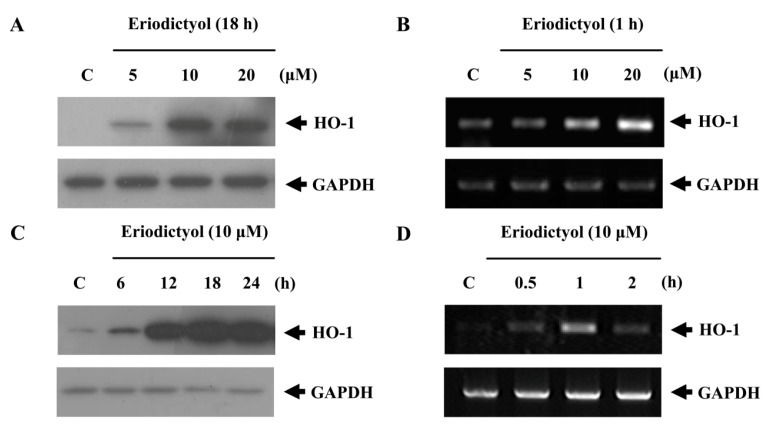

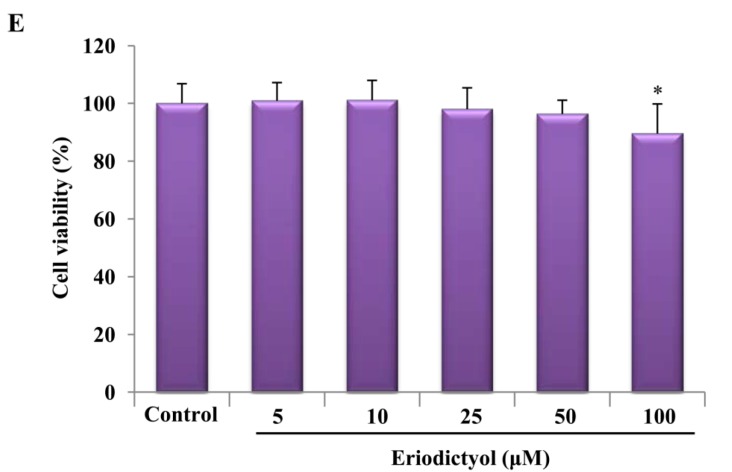
Upregulation of HO-1 by eriodictyol in HUVECs. Cells were treated with the indicated concentrations of eriodictyol (5, 10, and 20 μM) for 18 (**A**) and 1 h (**B**), and HO-1 levels were measured by Western blot and RT-PCR. GAPDH (glyceraldehyde 3-phosphate dehydrogenase) served as a loading control; (**C**,**D**) Cells were treated with 10 μM eriodictyol for the indicated times, and HO-1 levels were measured by Western blot and RT-PCR; and (**E**) Cell viability was estimated by MTT method. Data represents the mean ± SD of results in three independent experiments. * *p*  <  0.05 *vs*. control group.

The upregulation of HO-1 in cells is responsible for maintaining redox homeostasis and plays an essential role in protection against oxidative stress. In the present study, we assessed the effects of a range of concentrations of eriodictyol on HO-1 upregulation. Eriodictyol induced HO-1 protein and mRNA expression in a concentration-dependent manner (Figure 2A,B). Treatment with eriodictyol for 18 and 1 h markedly induced HO-1 protein and mRNA expression, respectively (Figure 2C,D).

Cell viability of HUVECs was evaluated by the MTT assay after 18 h of stimulation with various concentrations of eriodictyol (Figure 2E). At the concentrations used in this experiment, eriodictyol did not influence cell viability. These results suggest that concentrations of eriodictyol below 100 μM are not toxic to HUVECs. Therefore, in all of the experiments, cells were treated with eriodictyol at the concentration of 10 μM.

**Figure 3:**
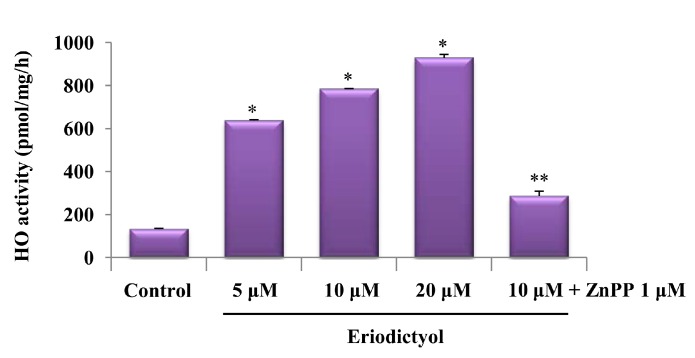
HO-1 activity in cells was measured 18 h after treatment with various concentrations of eriodictyol. Each bar represents the mean ± SD of four independent experiments. * *p*  <  0.05 *vs*. control group; ** *p*  <  0.05, between eriodictyol 10 μM and eriodictyol 10 μM plus ZnPP 1 μM co-treated samples.

After treatment with 5, 10, and 20 μM eriodictyol, its effects on HO-1 activity in endothelial cells were also observed. Exposure of the cells to eriodictyol for 18 h resulted in enhanced HO activity compared to control cells (Figure 3; untreated cells, * *p* < 0.05).

### 2.2. Blockage of Eriodictyol-Stimulated HO-1 Expression by ERK Inhibitor

**Figure 4:**
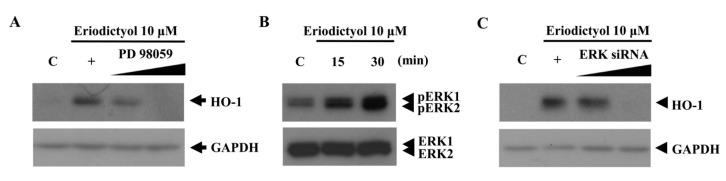
Blockage of eriodictyol-induced HO-1 protein expression by an ERK inhibitor. (**A**) Cells were pre-treated with increasing doses of PD 98059 (an ERK inhibitor) for 1 h prior to the treatment of eriodictyol 10 μM; (**B**) cell lysates were immunoblotted with antibodies against the phosphorylated form of ERK1/2 and total ERK; and (**C**) transient transfection of cells with increasing doses of ERK siRNA (20 and 30 nM) inhibited the induction of HO-1 protein expression by eriodictyol 10 μM. Western blots representative of three independent experiments are shown: C, untreated cells; +, treated with eriodictyol only; black line, treated with 10 µM eridictyol; 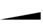, dose increasing.

Several signaling pathways have been reported to be involved in induction of HO-1 expression [23,24]. To verify the upstream signaling pathway involved in eriodictyol-induced HO-1 expression, the effects of specific inhibitors of the protein kinase C (PKC), p38 MAPK, ERK, and phosphatidylinositol 3 kinase (PI3K) pathways were assessed. Specifically, inhibitors of the ERK pathway considerably reduced eriodictyol-stimulated HO-1 expression (Figure 4A). To measure the activation of ERK, we detected increased phospho-ERK1/2 levels in eriodictyol-exposed cells (Figure 4B). Thus, we confirmed whether ERK signaling is involved in the upregulation of HO-1 expression by using siRNA against ERK. Introduction of scrambled siRNA had no effect on eriodictyol upregulation of HO-1 (data not shown). Compared to negative controls, ERK proteins declined after silencing with ERK siRNA (data not shown). In contrast, ERK siRNA restrained eriodictyol-stimulated HO-1 expression (Figure 4C). This observation supports a role for ERK signaling in eriodictyol-mediated HO-1 upregulation.

### 2.3. Eriodictyol Induced Nrf2 Nuclear Translocation and ARE-Luciferase Reporter Activity

**Figure 5:**
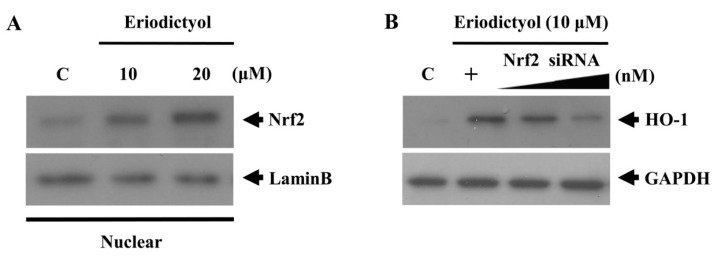
Nrf2 nuclear translocation induced by eriodictyol. (**A**) Cells were treated with 10 and 20 μM eriodictyol at the indicated concentrations for 4 h. Nuclear extracts were subjected to Western blot, using an anti-Nrf2 antibody and anti-lamin B antibody (a marker of nuclear protein); and (**B**) Transient transfection of cells with increasing doses of Nrf2-specific siRNA (10 and 20 nM) reduced HO-1 expression. Western blots representative of three independent experiments are shown: C, untreated cells; +, eriodictyol treatment only; 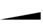, dose increasing.

Phase II antioxidant and detoxifying enzymes such as HO-1, are modulated by the Nrf2/Keap1 transcription factor system, which binds to the ARE in the nucleus [25]. Therefore, we observed whether eriodictyol could trigger Nrf2 nuclear translocation in association with the upregulation of HO-1. Treatment with eriodictyol showed an increase in the nuclear levels of Nrf2 (Figure 5A). We also determined the effect of knockdown of Nrf2 through siRNA transfection on eriodictyol-stimulated HO-1 expression. After silencing with Nrf2 siRNA, Nrf2 proteins were reduced in total cell lysates (data not shown), and the eriodictyol-stimulated enhancement of HO-1 was eliminated (Figure 5B), indicating that eriodictyol-stimulated HO-1 upregulation requires the activation of Nrf2.

**Figure 6:**
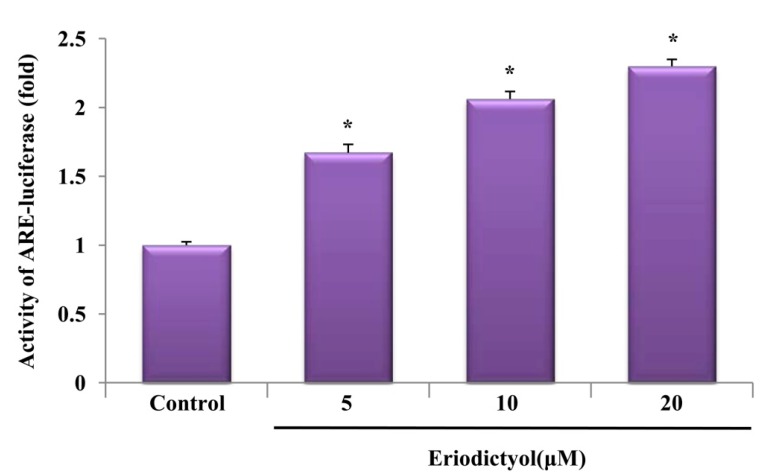
Activation of the ARE-luciferase reporter by eriodictyol. Cells were transfected with an ARE-luciferase construct. After transfection, the cells were treated with the indicated concentrations of eriodictyol for 6 h and the lysates were mixed with a luciferase substrate. A luminometer was used for measurement of luciferase activity. Data are presented as mean ± SD of quintuplicate experiments. * *p* < 0.05 *vs*. control.

In addition, we examined ARE promoter activity in eriodictyol-stimulated cells by using the luciferase reporter system. After transient transfection with the ARE luciferase reporter plasmids, cells were treated with 10 μM eriodictyol for 6 h, and luciferase activity was assessed. As expected, exposure to eriodictyol obviously amplified ARE promoter activity in a concentration-dependent manner (Figure 6, * *p* < 0.05). These results indicate that Nrf2/ARE is an essential transcription factor system for eriodictyol-induced HO-1 expression.

### 2.4. Protective Effect of Eriodictyol-Induced HO-1 against Oxidative Stress

**Figure 7:**
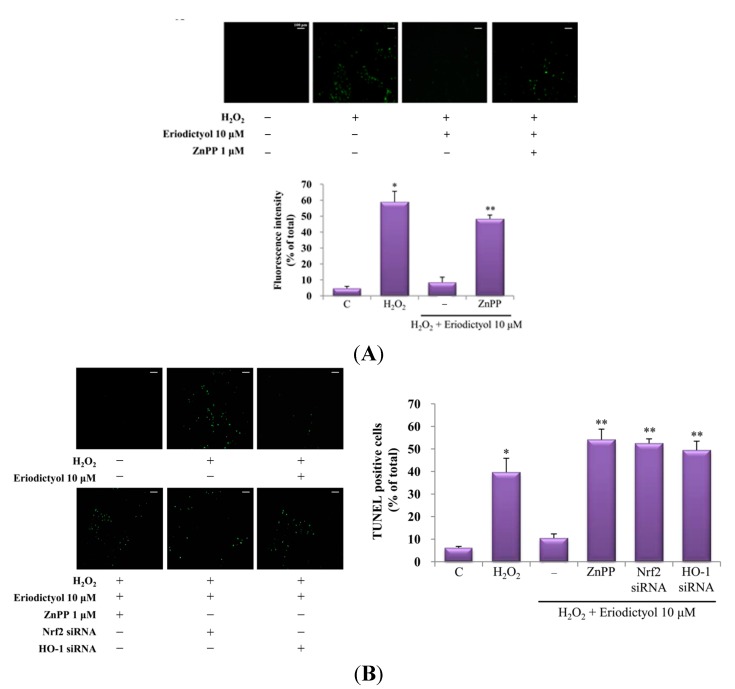
Protective effect of eriodictyol-induced HO-1 expression on H_2_O_2_-induced cell damage. (**A**) H_2_O_2_-exposed cells were pretreated for 1 h with or without ZnPP and then treated with eriodictyol. The inhibitory effect of eriodictyol on H_2_O_2_-induced intracellular ROS generation was observed by fluorescence microscopy; (**B**) H_2_O_2_-stimulated cells were pretreated for 1 h with or without ZnPP or Nrf2 or HO-1 siRNA and then treated with eriodictyol. Protective effect of HO-1 induction on cell death was determined by *in situ* terminal nick end-labeling (TUNEL) assay: −, untreated; +, treated. ROS and TUNEL staining was quantified in four randomly selected fields for each group: C, control. The scale bars for image (**B**) are the same as that in (**A**), 100 μm. * *p* < 0.05 *vs*. control, ** *p* < 0.05 *vs*. eriodictyol 10 μM + H_2_O_2_ co-treated samples.

Oxidative stress is considered a major factor in vascular disease, and an increase in intracellular reactive oxygen species (ROS) levels causes cellular dysfunction [26]. HO-1, a well-known cytoprotective enzyme, protects from cell damage induced by oxidative stress in diverse cell types [27,28,29]. To further elucidate whether the upregulation of HO-1 induced by eriodictyol confers cytoprotection, eriodictyol-exposed cells were pre-treated with or without a specific HO-1 inhibitor (zinc protoporphyrin, ZnPP) and the intracellular ROS level was assessed by DCF/DA staining. Pre-treatment with ZnPP showed augmented ROS production following exposure to eriodictyol and H_2_O_2_, compared to cells treated with eriodictyol and H_2_O_2_ alone (Figure 7A). As shown in Figure 3, HO-1 activity was augmented in the eriodictyol-exposed group, whereas pre-treatment with ZnPP notably decreased eriodictyol-enhanced HO-1 activity. These data imply that HO-1 upregulation by eriodictyol restrains ROS generation and that the cytoprotective effect of eriodictyol is mediated by the upregulation of HO-1.

Wang *et al.* reported that HO-1 could exert cytoprotective effect by preventing apoptosis [30]; therefore, we explored the association between the promotion of HO-1 activity and the cytoprotective effect of eriodictyol in oxidative stress-mediated cell death. For this purpose, we inhibited HO-1 enzymatic activity through treatment with a specific HO-1 inhibitor, ZnPP, or Nrf2/HO-1 siRNA and the presence of dead cells was assessed by *in situ* terminal nick end-labeling (TUNEL staining), which is widely used in detecting DNA fragmentation *in situ*. Treatment with eriodictyol illustrated a reduction in the proportion of TUNEL-positive cells, while inhibition of HO-1 (by treatment with ZnPP or transient transfection of HO-1 siRNA) or the transcription factor Nrf2 showed a prominent increase in the proportion of TUNEL-positive cells in H_2_O_2_ (300 μM)-exposed cells (Figure 7B). These results support that the upregulation of HO-1 by eriodictyol exerts a cytoprotective effect against oxidative stress-induced cell death.

## 3. Discussion

Vascular disease is the most common cause of deaths causing humans worldwide and is the leading cause of adult disability. Oxidative stress is a major risk factor in the pathogenesis of vascular disorder, suggesting that therapeutic strategies directed against oxidative stress are particularly valuable. Recently, increasing evidence has shown that the induction of the antioxidant enzymes by polyphenols can confer protection against vascular injury. Several studies highlight the ability of these natural plant-derived compounds to protect against vascular disease through activation of the antioxidant enzymes, such as HO-1 [30,31,32].

Moreover, many studies have supported the beneficial association between health and polyphenols in fruits and vegetables. Among the various polyphenols, flavonoids in particular exhibit various biological properties, including anti-cancer, anti-inflammatory, and anti-oxidative effects. A range of health benefits related to the Mediterranean diet has been attributed to flavonoids such as protection from cancer and cardiovascular disease [33,34]. Eriodictyol, a flavonoid present in fruits and vegetables, has long been established as a potent antioxidant. Recently, numerous studies have demonstrated that eriodictyol could serve as a pharmaceutical, nutraceutical, or functional food for patients with a range of disorders such as atopic dermatitis and diabetes [35,36,37,38]. Nevertheless, the effects and underlying mechanisms of HO-1 upregulation by eriodictyol have not been explored in endothelial cells. In this study, we discovered that the flavonoid eriodictyol clearly induces HO-1 expression through ERK/Nrf2/ARE-dependent pathways in endothelial cells.

The upregulation of phase II enzymes by natural compounds such as flavonoids exhibits protective effects [39,40]. Among phase II cytoprotective enzymes, HO-1, a stress-inducible protein, is broadly distributed in mammalian tissues. HO-1 has anti-inflammatory and antioxidant effects that result in protective actions in various pathological models [41,42,43]. Moreover, the promotion of HO-1 activity protects against oxidative stress-induced cell death [44,45].

Numerous kinases and Nrf2/ARE-dependent transcription are known to be involved in HO-1 expression. The present study revealed that the ERK pathway and the translocation of Nrf2 in the nucleus are required for eriodictyol-induced HO-1 expression. Additionally, the effect of Nrf2 knockdown on HO-1 expression supports the assertion that the upregulation of HO-1 is associated with the transcription factor Nrf2. In response to stimuli, Nrf2 separates from its inhibitory protein, Kelch-like ECH-associated protein 1 (Keap1), and translocates to the nucleus, wherein it binds to the ARE sequence to activate transcription of a variety of anti-oxidative and detoxification genes such as HO-1 [25]. Increase in ARE promoter activity by eriodictyol treatment indicates that it could enhance ARE transcriptional activity. Collectively, these data imply that eriodictyol-stimulated HO-1 expression requires activation of ERK/Nrf2/ARE in endothelial cells.

The upregulation of HO-1 inhibits oxidative stress-induced cell death [6]. In the present study, we examined that suppression of eriodictyol-induced HO-1 expression by treatment with the HO-1 inhibitor or Nrf2 or HO-1 siRNA increased cell death in H_2_O_2_-stimulated endothelial cells, suggesting that eriodictyol-induced HO-1 expression serves as a mechanism for protecting cells against oxidative stress.

In summary, we demonstrated that eriodictyol, a naturally-derived flavonoid, up-regulates HO-1 expression in human primary endothelial cells in association with ERK/Nrf2/ARE signaling pathways, and it can potently diminish H_2_O_2_-induced oxidative damage via the induction of HO-1. Our findings indicate that eriodictyol has potential therapeutic application in protecting against oxidative stress-related diseases, including cardiovascular disease.

## 4. Experimental Section

### 4.1. Materials

Eriodictyol, 3-(4,5-dimethylthiazol-2-yl)-2,5-diphenyltetrazoliumbromide (MTT), dimethylsulphoxide, and zinc protoporphyrin (ZnPP) were obtained from Sigma (St. Louis, MO, USA). M199 medium and fetal bovine serum were purchased from WelGENE (Daegu, Korea). TRIzol reagent was supplied by Invitrogen (Carlsbad, CA, USA). ExGenTM 500 reagent was obtained from Fermentas (Hanover, MD, USA). TransPass R2 Transfection Reagent was supplied by New England Biolabs (Hercules, CA, USA). The following antibodies were used: anti-Nrf2 (Santa Cruz Biotechnology, Santa Cruz, CA, USA), anti-lamin B (Santa Cruz, CA, USA), anti-HO-1 (Epitomics, Burlingame, CA, USA), anti-ERK1/2 (Cell Signaling Technology, Beverly, MA, USA), and anti-GAPDH (glyceraldehyde 3-phosphate dehydrogenase) (AbFrontier, Seoul, Korea). PD98059, SB203580, rottlerin, LY294002, and SP600125 were purchased from Calbiochem (La Jolla, CA, USA). Nrf2 (SC-37049) siRNAs were obtained from Santa Cruz Biotechnology, and ERK siRNA was purchased from Bioneer (Daejeon, Korea). All other chemicals and reagents used were of analytical grade.

### 4.2. Cell Culture and Viability Measurement

HUVECs (StemCell Technologies, Vancouver, BC, Canada) were maintained in M199 medium supplemented with 10% fetal bovine serum, 100 U/mL penicillin, 100 µg/mL streptomycin, 10 ng/mL human fibroblast growth factor, and 18 mU/mL heparin, at 37 °C under an atmosphere of 5% CO_2_. The cells were grown to approximately 80% confluence, maintained using fresh culture medium, and subcultured every 2−3 days. The cells were used within passages 4−9 during these experiments.

Cell viability was measured using the conventional MTT assay as described [28].

### 4.3. Western Blot Analysis

After eriodictyol treatment, cells were washed with phosphate-buffered saline and mixed with RIPA (50 mM Tris CL, pH 7.4/150 mM NaCl/1% Nonidet P-40 (NP-40)/1% sodium deoxycholate/0.1% SDS) buffer containing 1 mM etilendiaminetetraacetic acid (EDTA), 5 µg/mL aprotinin, 2 µg/mL leupeptin, and 1 mM phenylmethylsulfonyl fluoride (PMSF), followed by centrifugation at 14,000× *g* for 15 min. We applied 20 μg of the whole cell lysate protein to each lane and analyzed them by Western blot, using a monoclonal antibody against HO-1, anti-Nrf2, anti-Lamin B, and GAPDH). Horseradish peroxidase-conjugated anti-IgG antibodies were used as the secondary antibodies to detect the abovementioned protein bands by enhanced chemiluminescence WESTSAVE-Up ECL kit (AbFrontier, Seoul, Korea).

### 4.4. RNA Isolation and Reverse Transcriptase-Polymerase Chain Reaction

Reverse transcription was performed as described previously [28]. The primer sequence for human HO-1 was 5′-ACATCTATGTGGCCCTGGAG-3′ (forward) and 5′-TGTTGGGGAAGGTGAAGAAG-3′ (reverse) [28]. The amplified products were resolved by 1% agarose gel electrophoresis, stained with ethidium bromide, and photographed under ultraviolet light.

### 4.5. Assay for HO Activity

HO enzyme activity was measured as described previously [28]. Briefly, microsomes from harvested cells were added to a reaction mixture containing NADPH, rat liver cytosol as a source of biliverdin reductase, and hemin as the substrate. The reaction was carried out in the dark for 1 h at 37 °C, and the amount of extracted bilirubin was determined by calculating difference in absorbance at 464 and 530 nm.

### 4.6. Nrf2 and ERK Silencing by siRNA

HUVECs were plated in 6-well plates at a density of 2.0 × 10^5^ cells per well and transfected with ERK or Nrf2-siRNA or scrambled siRNA for 18 h. For each transfection, 1200 μL of the transfection medium was was added to 0.25–1 μg or 10–30 nM of the siRNA duplex/transfection reagent mix (TransPass R2 solution A + B), and the entire volume was added gently to the cells.

### 4.7. Preparation of Nuclear Proteins

Cells were incubated with various concentrations of eriodictyol for 4 h, and were then washed with PBS and centrifuged at 3300× *g* for 5 min at 4 °C. Pellets were resuspended in ice-cold isotonic buffer A (10 mM 4-(2-hydroxyethyl)-1-piperazineethanesulfonic acid (HEPES) (pH 7.9), 10 mM KCl, 0.1 mM EDTA, 1 mM dithiothreitol (DTT), 0.5 mM phenylmethylsulfonyl fluoride (PMSF)), and a protease inhibitor cocktail containing 0.3 μM aprotinin and 2 mM leupeptin. After 15 min of incubation in an ice bath, cells were vortexed vigorously for 10 s with addition of 10% NP-40 and recentrifuged at 7000× *g* for 2 min at 4 °C. Pellets were resuspended in ice-cold buffer B containing 20 mM HEPES (pH 7.9), 0.4 M NaCl, 1 mM EDTA, 10% glycerol, 1 mM DTT, 1 mM PMSF, and the protease inhibitor cocktail, followed by incubation at 4 °C for 30 min with periodic vortexing. The mixture was then centrifuged at 12,000× *g* for 30 min at 4 °C. The supernatant was collected and stored −70 °C for protein assay and Western blot analysis.

### 4.8. Measurement of ARE Promoter Activity

The EpRE/ARE-luciferase (EpRE/ARE-Luc) reporter plasmid was a generous gift from Rae-Kil Park (Wonkwang University, Iksan, Korea). The plasmid was generated by transfer of the enhancer 2 (E2) and minimal promoter (MP) sequences into the luciferase reporter plasmid pGL3-Basic [46]. Promoter activity was determined as described previously [44].

### 4.9. Measurement of Intracellular Reactive Oxygen Species (ROS) Generation 

Intracellular ROS in hydrogen peroxide (H_2_O_2_)-stimulated cells was analyzed by staining using DCF/DA. In brief, 4.0 × 10^5^ cells were incubated in 60 mm-diameter dishes with or without 1 μM ZnPP for 1 h. The cells were treated with eriodictyol or vehicle for 1 h followed by treatment of the cells with 300 μM H_2_O_2_ for 17 h. Then, the cells were stained with 10 μM DCF/DA for 60 min. After rinsing with phosphate-buffered saline, the cells were examined by fluorescence microscopy.

### 4.10. Terminal Deoxynucleotidyl Transferase-Mediated dUTP Nick End-Labeling (TUNEL) Assay

DNA fragmentation was measured using the commercially available In Situ Cell Death Detection kit (Roche Diagnostics, Mannheim, Germany). HUVECs were cultured in a glass culture chamber slide and fixed for 30 min in a 10% neutral buffered formalin solution at room temperature. A TUNEL assay system was used, according to the manufacturer’s instructions, for examination under a fluorescence microscope (Eclipse 50i, Nikon, Tokyo, Japan), with excitation at 488 nm and emission at 525 nm.

### 4.11. Statistical Analysis

Statistical significance was estimated using one-way analysis of variance (ANOVA) followed by Bonferroni’s *post-hoc* test. The results are expressed as mean ± standard deviation (S.D).
